# CCL2-Mediated Stromal Interactions Drive Macrophage Polarization to Increase Breast Tumorigenesis

**DOI:** 10.3390/ijms24087385

**Published:** 2023-04-17

**Authors:** Maddison Archer, Sarah M. Bernhardt, Leigh J. Hodson, Lucy Woolford, Mark Van der Hoek, Pallave Dasari, Andreas Evdokiou, Wendy V. Ingman

**Affiliations:** 1 Discipline of Surgical Specialties, Adelaide Medical School, University of Adelaide, The Queen Elizabeth Hospital, Woodville South, SA 5011, Australia; 2 Robinson Research Institute, University of Adelaide, Adelaide, SA 5006, Australia; 3 School of Animal and Veterinary Sciences, Faculty of Sciences, Roseworthy Campus, University of Adelaide, Roseworthy, SA 5371, Australia; 4 South Australian Genomics Centre, South Australian Health and Medical Research Institute, Adelaide, SA 5000, Australia

**Keywords:** mammographic density, breast tumorigenesis, immune signalling, inflammation, fibroblasts, macrophages

## Abstract

CCL2 is an inflammatory cytokine that regulates macrophage activity and is implicated in increased mammographic density and early breast tumorigenesis. The role of CCL2 in mediating stromal interactions that contribute to breast tumorigenesis has yet to be fully elucidated. THP-1-derived macrophages and mammary fibroblasts were co-cultured for 72 h. Fibroblasts and macrophages were analysed for phenotype, expression of inflammatory and ECM-regulatory genes and collagen production. Mice overexpressing CCL2 in the mammary glands were analysed for global gene expression by RNAseq at 12 weeks of age. These mice were cross-bred with PyMT mammary tumour mice to examine the role of CCL2 in tumorigenesis. The co-culture of macrophages with fibroblasts resulted in macrophage polarization towards an M2 phenotype, and upregulated expression of CCL2 and other genes associated with inflammation and ECM remodelling. CCL2 increased the production of insoluble collagen by fibroblasts. A global gene expression analysis of CCL2 overexpressing mice revealed that CCL2 upregulates cancer-associated gene pathways and downregulates fatty acid metabolism gene pathways. In the PyMT mammary tumour model, CCL2 overexpressing mice exhibited increased macrophage infiltration and early tumorigenesis. Interactions between macrophages and fibroblasts regulated by CCL2 can promote an environment that may increase breast cancer risk, leading to enhanced early tumorigenesis.

## 1. Introduction

C-C Chemokine Ligand 2 (CCL2) is a cytokine that acts as a chemoattractant for monocytes and macrophages to sites of inflammation [1]. The expression of CCL2 is largely induced by oxidative stress or cell activation by growth factors and cytokines [2]. CCL2 is expressed by several different cell types including fibroblasts, epithelial cells, smooth muscle cells, and endothelial cells [3,4]. The main cellular target of CCL2 is macrophages and the primary receptor, C-C Chemokine Receptor type 2 (CCR2), is expressed on leukocytes, monocytes and macrophages.

CCL2 has been implicated in tumour development, and its expression by tumour cells is associated with poor prognosis in women with breast cancer [5,6]. CCL2 promotes the recruitment of tumour-associated macrophages (TAMs) and increases the invasive properties of cancer cells, thereby promoting metastasis to the lungs and bone [5,6,7,8]. A key role for CCL2 in early breast tumorigenesis is through increasing mammographic breast density, which is an independent risk factor for breast cancer [9,10]. Mice that constitutively express CCL2 in the mammary gland exhibit increased macrophage abundance and increased stromal density compared to wildtype controls [11], both of which are biological characteristics of high mammographic density [12,13]. When challenged with a chemical carcinogen, CCL2 overexpressing mice had increased mammary cancer susceptibility, with decreased tumour latency and decreased tumour-free survival [11]. In humans, an analysis of paired high and low mammographic density tissue samples demonstrated that elevated CCL2 expression is associated with high mammographic density [11]. While CCL2 is associated with increased early breast tumorigenesis, how CCL2-regulated macrophages interact with other cell components within the breast stroma has not been investigated.

Fibroblasts are an abundant cell type in high mammographic density breast tissue; however, they are unlikely to have an independent role in the promotion of breast cancer risk associated with high density [14]. In vitro studies of primary mammary fibroblasts derived from high or low density tissue demonstrated that their activity is affected by immune mediators, suggesting that immune cells, such as macrophages, in the breast could be part of the regulatory mechanism that determines the abundance of stromal fibroblasts and the extracellular matrix (ECM) that is a key feature of high mammographic density. Indeed, macrophages are in greater abundance in breast tissue from regions of high density compared to regions of low density [15]. 

Macrophages have diverse roles in the regulation of mammary gland development and tumorigenesis. Regulated by cytokines and hormones in the mammary gland microenvironment [16,17,18], macrophages promote the proliferation of epithelial cells and tissue remodelling [19,20]. They also play a role in breast tumorigenesis, with macrophage infiltration associated with poor prognosis in breast cancer patients [21]. Macrophages exert a multitude of functions in cancer, and are often described as polarized to either the M1 or M2 phenotype depending on the expression of specific markers on their cell surface. Macrophages identified as the M2 phenotype promote mammary tumour progression by immune suppression, tumour growth, metastasis and angiogenesis [21,22,23]. On the other hand, macrophages identified as the M1 phenotype protect against cancer through immune surveillance functions and production of inflammatory signals that facilitate anti-tumour immune responses [24,25]. However, this dichotomy is an over-simplification and it is clear that the central role macrophages play in tissue homeostasis involves a more complex relationship between how they sense their environment and the impact of this on their function [26]. For example, CCL2-mediated inflammation is associated with M2 polarized macrophages and tumorigenesis in colorectal cancer [27]. Therefore, it is important to consider macrophage polarization in the context of the presence and function of other cell types within the specific tissue microenvironment.

Macrophages and fibroblasts exist together within the breast stroma and cell-to-cell interactions between these cell types may affect the abundance of stroma and ECM. Macrophages regulate ECM turnover by producing enzymes that promote and/or inhibit collagen break down such as matrix metalloproteinases (MMPs), tissue inhibitors of metalloproteinases (TIMPs), as well as other growth factors [28,29]. Macrophages can also influence tissue remodelling by regulating the production of collagen by fibroblasts [30,31,32]. Interactions between fibroblasts and macrophages are also significant in shaping the tumour microenvironment in cancer. Cancer-associated fibroblasts (CAFs) recruit macrophages to the tumour site through the expression of chemoattractant signals such as CCL2 [33,34] and regulate the immune response to tumour cells by driving the polarization of macrophages with pro- or anti-tumorigenic phenotypes [35,36]. Similarly, the expression of soluble factors from macrophages can induce the expression of CAF markers in fibroblasts [34,37].

Currently, how macrophages interact with surrounding stromal cells such as fibroblasts in the breast and how this may contribute to CCL2-mediated mammographic density and early breast tumorigenesis has not been fully elucidated. Here, we explore macrophage–fibroblast interactions that regulate CCL2 ligand and receptor expression and ECM deposition, and how CCL2 affects tumorigenesis using a mouse model of breast cancer.

## 2. Results

### 2.1. The Effect of Mammary Fibroblasts on THP-1-Derived Macrophage Phenotype in Co-Culture

**Figure 1:**
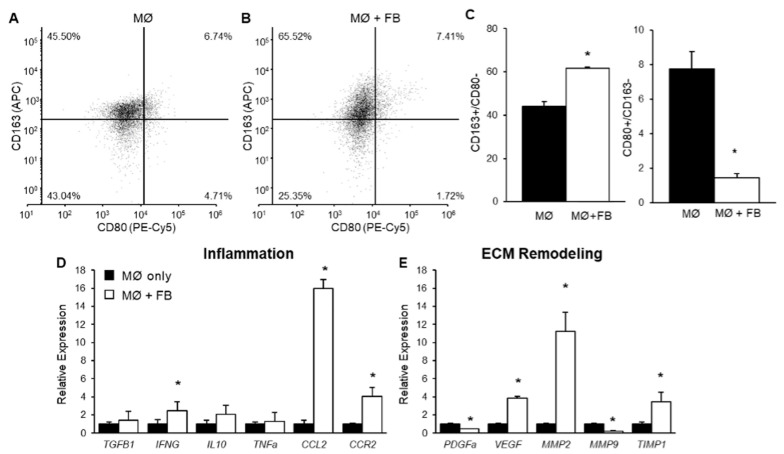
THP-1-derived macrophage phenotype following indirect co-culture with mammary fibroblasts. PMA-treated THP-1 derived macrophages (0.5 × 10^6^ cells/mL) were cultured alone (MØ only), or indirectly with primary mammary fibroblasts (FB + MØ) for 72 h. Cell surface marker expression of CD163 and CD80 on THP-1 derived macrophages were detected using flow cytometry in (**A**) macrophage only cultures (*n* = 6) and (**B**) macrophages in indirect co-culture with primary mammary fibroblasts (*n* = 6). (**C**) The percentage of viable cells, which are CD163^+^/CD80^−^ or CD80^+^/CD163^−^ in each culture condition. Expression of genes associated with (**D**) inflammatory and (**E**) extracellular matrix (ECM) remodelling were assessed through RT-PCR. Gene expression was normalised to the housekeeping gene HPRT1. Results are presented relative to macrophage-only cultures. Data presented as mean + SEM. Statistical analysis was performed using a linear mixed effects model with Tukey’s post hoc comparison. Statistical significance is indicated by * when *p* < 0.05.

To investigate the effect of mammary fibroblasts on THP-1-derived macrophages, PMA-treated macrophages were co-cultured with primary mammary fibroblasts for 72 h. The macrophages were then analysed by flow cytometry to determine the macrophage phenotype. The macrophage phenotype was determined by the expression of surface markers CD80 and CD163. M0 cells were classified as CD80^−^/CD163^−^ cells, M1 macrophages were classified as CD80^+^/CD163^−^ cells, and M2 macrophages were classified as CD163^+^/CD80^−^ negative cells. These markers are consistently used in the literature [38,39,40]. PMA-treated macrophages alone in culture were approximately 40% M2 phenotype and 8% M1 phenotype, with the remainder in M0 (Figure 1A). The population of M1 CD80^+^/CD163^−^ macrophages was significantly decreased in THP-1-derived macrophages in co-culture with mammary fibroblasts (1.45 ± 0.24%) compared to macrophage only controls (7.75 ± 1.00%) (*p* < 0.01) (Figure 1B,C). The population of M2 CD163^+^/CD80^−^ macrophages was significantly increased from 44.03 ± 2.3% in macrophage only controls to 61.69 ± 0.67% in macrophages co-cultured with mammary fibroblasts (*p* < 0.01) (Figure 1B,C). 

### 2.2. The Effect of Mammary Fibroblasts on THP-1 Derived Macrophage Gene Expression

To investigate the effects of primary mammary fibroblasts on THP-1 derived macrophage gene expression, PMA-treated THP-1 cells were indirectly co-cultured with primary mammary fibroblasts for 72 h (*n* = 6). The mRNA expression of cytokines *TGFB1*, *IL10*, *IFNG* and *TNFA* was detected by RT-PCR as well as expression of the CCL2 receptor *CCR2*. Genes involved in tissue remodelling and growth factors such as *MMP2*, *MMP9*, *TIMP1*, *PDGF* and *VEGF* were also measured using quantitative RT-PCR relative to the expression of housekeeping gene *HPRT1* from the same patient. The results are expressed in arbitrary units, were the data were normalised so that the average expression of each gene in the untreated controls was equal to 1 (Figure 1D,E).

It was observed that *IFNG* mRNA expression was significantly increased 4-fold in macrophages co-cultured with mammary fibroblasts (3.92 ± 0.78 arbitrary units) compared to macrophage only controls (1.00 ± 0.49 arbitrary units) (*p* < 0.05). The expression of *CCL2* by macrophages was significantly increased 15-fold in co-culture with mammary fibroblasts. The expression of mRNA encoding the CCL2 receptor *CCR2* was increased 4-fold in fibroblast co-cultured THP-1 derived macrophages in the (4.02 ± 0.72 arbitrary units) compared to macrophage only controls (1.00 ± 0.11 arbitrary units) (Figure 1D).

The expression of mRNA encoding MMP2 was significantly increased 11-fold in co-cultured THP-1-derived macrophages (11.23 ± 2.11 arbitrary units) compared to macrophage only controls (1 ± 0.10 arbitrary units) (*p* < 0.01) (Figure 1E). The expression of mRNA-encoding MMP9 was significantly decreased in THP-1-derived macrophages when co-cultured with mammary fibroblasts (0.22 ± 0.04 arbitrary units) compared to macrophage only controls (1.00 ± 0.12 arbitrary units) (*p* < 0.05). The expression of mRNA-encoding TIMP1 was significantly increased 3-fold in co-cultured macrophages (3.48 ± 1.00 arbitrary units) compared to macrophage only controls (1.00 ± 0.20 arbitrary units) (*p* < 0.01). A reduction in expression of mRNA-encoding PDGF was observed in co-cultured macrophages compared to macrophage only controls (*p* < 0.05) (Figure 1E). It was observed that mRNA expression of *VEGF* was significantly increased 4-fold in THP-1-derived macrophages when co-cultured with mammary fibroblasts (3.87 ± 0.15 arbitrary units) compared to macrophage only controls (1.00 ± 0.05 arbitrary units) (*p* < 0.05) (Figure 1E).

### 2.3. The Effect of THP-1 Derived Macrophages on Mammary Fibroblast Gene Expression in Co-Culture

To investigate the effects of THP-1-derived macrophages on primary mammary fibroblast gene expression, PMA-treated THP-1 cells were indirectly co-cultured with primary mammary fibroblasts for 72 h (*n* = 6). The expression of mRNA-encoding genes involved in inflammation, growth factor signalling, ECM remodelling and matrix metalloproteinases were measured by RT-PCR. The data presented are the relative expression to the housekeeping gene *HPRT1* for each culture condition, and the results were normalised so the average expression of fibroblast only controls is 1 arbitrary unit. 

**Figure 2:**
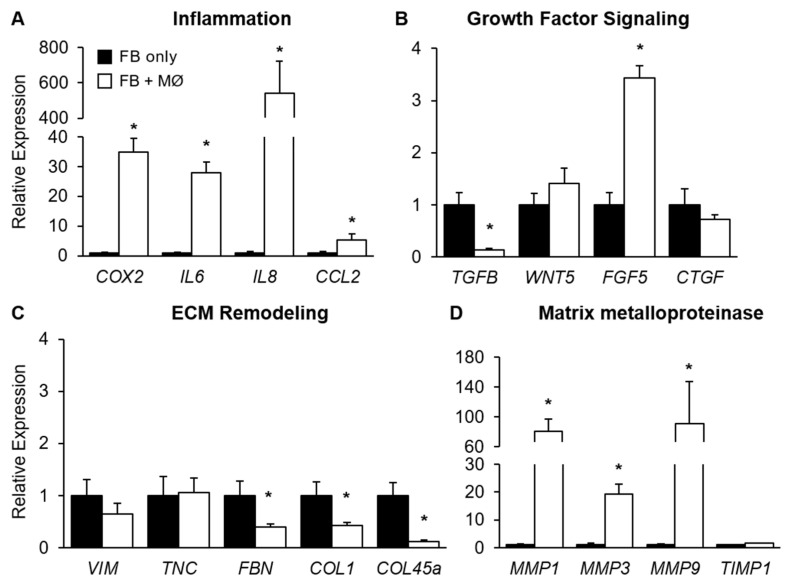
Gene expression in mammary fibroblasts following indirect co-culture with THP-1 derived macrophages. Primary mammary fibroblasts were cultured alone (FB only), or indirectly with PMA-treated THP-1-derived macrophages (0.5 × 10^6^ cells/mL) (FB + MØ) for 72 h. Expression of genes associated with (**A**) inflammation, (**B**) growth factor signalling, (**C**) extracellular matrix remodelling, and (**D**) matrix metalloproteinase function were assessed through RT-PCR. Gene expression was normalised to the housekeeping gene HPRT1. Results are presented relative to fibroblasts-only cultures. All data mean + SEM. Statistical analysis was performed using a linear mixed effects model with Tukey’s post hoc comparison. Statistical significance is indicated by * when *p* < 0.05.

Marked changes in mRNA expression of inflammatory genes were observed in mammary fibroblasts when indirectly co-cultured with THP-1-derived macrophages. The expression of mRNA-encoding COX2 was significantly increased by 35-fold in fibroblasts when co-cultured with THP-1-derived macrophages (34.79 ± 4.73 arbitrary units) compared to fibroblast only controls (1.00 ± 0.28 arbitrary units) (*p* < 0.01). The expression of mRNA-encoding IL6 was significantly increased approximately 30-fold in fibroblasts co-cultured with THP-1-derived macrophages (28.04 ± 3.55 arbitrary units) compared to fibroblast only controls (1.00 ± 0.26 arbitrary units) (*p* < 0.01) (Figure 2A). The expression of mRNA-encoding IL8 was highly increased in mammary fibroblasts in co-culture with THP-1 macrophages with an over 500-fold increase (541.9 ± 180.6 arbitrary units) in expression compared to fibroblast only controls (1.00 ± 0.52 arbitrary units) (*p* < 0.01) (Figure 2A). The expression of CCL2 was significantly increased in mammary fibroblasts when co-cultured with THP-1-derived macrophages (*p* < 0.05) (Figure 2A).

The expression of *FGF5* mRNA was increased 3-fold in mammary fibroblasts in co-culture with THP-1-derived macrophages (3.43 ± 0.24 arbitrary units) compared to fibroblast only controls (1.00 ± 0.24 arbitrary units) (*p* < 0.01) (Figure 2B). In mammary fibroblasts in co-culture with THP-1-derived macrophages, the mRNA expression of *TGFB* was significantly decreased by approximately 85% (0.13 ± 0.03) compared to fibroblast only controls (1.00 ± 0.23 arbitrary units) (*p* < 0.01) (Figure 2B). The co-culture of primary mammary fibroblasts with THP-1-derived macrophages did not have any significant effects on mRNA expression on *WNT5* and *CTGF* (Figure 2B).

It was observed that the average mRNA expression of *FBN* was decreased by approximately 60% in fibroblasts when co-cultured with THP-1-derived macrophages (0.39 ± 0.07 arbitrary units), compared to fibroblast only controls (1.00 ± 0.28 arbitrary units) (*p* < 0.05). The expression of mRNA-encoding COL1A1 was observed to be decreased by 60% in primary mammary fibroblasts when co-cultured with THP-1-derived macrophages (0.43 ± 0.06 arbitrary units) compared to fibroblast only controls (1.00 ± 0.26 arbitrary units) (*p* < 0.01) (Figure 2C). In mammary fibroblasts co-cultured with THP-1-derived macrophages, mRNA expression of *COL4A5* was significantly decreased by 90% (0.13 ± 0.03 arbitrary units) compared to fibroblasts alone (1.00 ± 0.25 arbitrary units) (*p* < 0.01) (Figure 2C).

In mammary fibroblasts in co-culture with THP-1-derived macrophages, mRNA expression of *MMP1* was significantly increased by approximately 80-fold (80.42 ± 16.69 arbitrary units) compared to fibroblast only controls (1.00 ± 0.33 arbitrary units) (*p* < 0.01) (Figure 2D). The expression of mRNA-encoding MMP3 was also significantly increased in co-cultured mammary fibroblasts, by approximately 19-fold (19.21 ± 4.07 arbitrary units) compared to fibroblast only controls (1.00 ± 0.59 arbitrary units) (*p* < 0.01) (Figure 2D). A significant 90-fold increase in mRNA expression-encoding MMP9 was observed in mammary fibroblasts co-cultured with THP-1-derived macrophages (0.87 ± 56.58 arbitrary units) compared to fibroblast only controls (1.00 ± 0.46 arbitrary units) (*p* < 0.05). In mammary fibroblasts in co-culture with THP-1-derived macrophages, the expression of mRNA-encoding TIMP1 was significantly increased by approximately 60% (1.57 ± 0.16 arbitrary units) compared to fibroblast only controls (1.00 ± 0.23 arbitrary units) (*p* < 0.05) (Figure 2D).

### 2.4. The Effect of CCL2 on Mammary Fibroblast Collagen Production in Co-Culture

**Figure 3:**
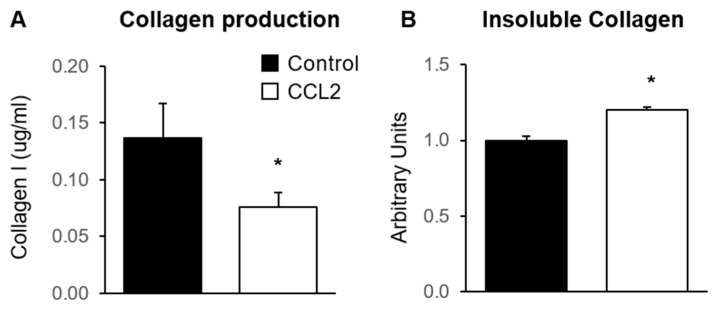
The effect of CCL2 on collagen production by mammary fibroblasts. Primary mammary fibroblasts were cultured alone (Control) or with 500 ng/mL of CCL2 for 72 h. Collagen was measured using collagen 1 ELISA that detects soluble collagen (**A**) and insoluable collagen was measured by Sirius Red staining (**B**). All data are presented as mean + SEM. Statistical analysis was performed using a linear mixed effects model with Tukey’s post hoc comparison. Statistical significance is indicated by * when *p* < 0.05.

To investigate the effect of CCL2 on collagen production in primary mammary fibroblasts, fibroblasts were cultured in the presence or absence of 500 ng/mL of CCL2 for 72 h (*n* = 6). Collagen was measured by ELISA to detect production of soluble collagen I, and Sirius red dye was used to measure insoluble collagen fibres (*n* = 6). The results were adjusted to be relative to fibroblast only controls, where the average level of collagen production was equal to 1 arbitrary unit. Collagen I production was significantly decreased in fibroblasts treated with CCL2 (0.56 ± 0.09 µg/mL) compared to untreated controls (1 ± 0.22 µg/mL) (*p* < 0.05) (Figure 3A). However, the abundance of insoluble collagen fibres, as measured with Sirius red dye, was significantly increased in fibroblasts treated with CCL2 (1.21 ± 0.07 arbitrary units) compared to untreated controls (1 ± 0.08 arbitrary units) (*p* < 0.05) (Figure 3B).

### 2.5. The Effect of CCL2 on Tumour Development in PyMT Mammary Tumour Models

**Figure 4:**
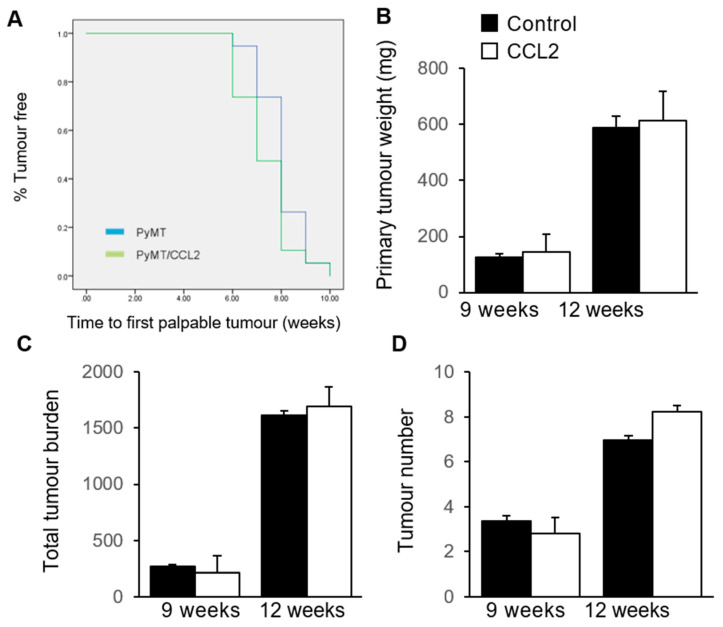
The effect of CCL2 overexpression on tumour development in PyMT transgenic mice. PyMT and PyMT/CCL2 mice were palpated weekly to monitor tumour latency from 6 weeks of age (*n* = 28 per group). (**A**) Kaplan–Meier survival plot of the percentage of tumour free mice for each week of monitoring. Primary and secondary tumours were collected at 9 (*n* = 9 per group) and 12 weeks of age (*n* = 10 per group). (**B**) Primary tumour weight, (**C**) total tumour burden (mg), and (**D**) number of tumours were measured in each mouse. Data presented as mean + SEM. Statistical analysis performed using SPSS, mean survival was tested using Log Rank test.

To investigate the effect of CCL2 on tumour latency in the Mmtv-PyMT mouse model, PyMT controls and PyMT/CCL2 mice were monitored for the emergence of palpable tumours weekly from 6 weeks of age, and sacrificed at either 9 or 12 weeks of age for analysis. A Kaplan–Meier plot was constructed to analyse the percentage of mice that were tumour-free in each week of monitoring. By 10 weeks of age, 100% of the mice had developed tumours. The average tumour latency of the PyMT controls and PyMT/CCL2 mice was 8 and 7.5 weeks, respectively (Figure 4A). There was no significant difference in tumour latency between groups as determined using SPSS Log Rank (*n* = 19 mice per group).

At the time of sacrifice, both the primary tumour and all other tumours were dissected and weighed to investigate the tumour burden. At 9 weeks of age, the average primary tumour weight was 124.28 ± 15.48 mg for PyMT controls and 145.33 ± 41.62 mg in PyMT/CCL2 mice. At 12 weeks of age, the average primary tumour weight was 588.39 ± 64.30 mg in PyMT control mice and 613.29 ± 104.01 mg in PyMT/CCL2 mice. There was no significant difference in primary tumour weight between PyMT/CCL2 and PyMT control mice at 9 (*p* = 0.586) and 12 (*p* = 0.885) weeks of age (Figure 4B). The total tumour burden was measured as the total weight of all mammary tumours in each mouse. The average total tumour burden at 9 weeks of age was 268.67 ± 19.04 mg for PyMT controls and 211.93 ± 45.43 mg for PyMT/CCL2 mice. The average total tumour burden at 12 weeks of age was 1611.79 ± 154.57 mg for PyMT controls and 1695 ± 169.46 mg in PyMT/CCL2 mice. There was no significant difference in the total tumour burden between PyMT/CCL2 and PyMT control mice at 9 (*p* = 0.187) and 12 (*p* = 0.706) weeks of age (Figure 4C). The average number of tumours in PyMT control mice was 3.36 ± 0.26 tumours and in PyMT/CCL2 mice, there were 2.8 ± 0.21 tumours at 9 weeks of age. At 12 weeks of age, the average number of tumours in PyMT control mice was 6.95 ± 0.73 tumours, and in PyMT/CCL2 mice there were 8.24 ± 0.26 tumours. There was no significant difference in the tumour number between PyMT and PyMT/CCL2 mice at 9 (*p* = 0.187) and 12 (*p* = 0.364) weeks of age (Figure 4D).

Primary tumours were classified into four tumour grades according to their morphology; hyperplasia, adenoma, early carcinoma and late carcinoma [41]. At 9 weeks of age, 100% of the primary tumours from both PyMT and PyMT/CCL2 mice were in the late carcinoma stage. In the 12-week-old cohort, 83% of the primary tumours collected from PyMT mice were late carcinoma (15 of 18 mice), with the remaining three being early carcinoma stage. In PyMT/CCL2 mice, 89% were late carcinoma (17 of 19 mice) and the remaining two were early carcinoma stage. There were no statistically significant differences in tumour grade between PyMT control mice and PyMT/CCL2 mice. No differences were observed in cytological atypia or tumour necrosis in primary tumours from PyMT control mice and PyMT/CCL2 mice. No differences were observed in the incidence of pulmonary metastasis in 12-week-old PyMT control mice and PyMT/CCL2 mice. Lung metastases were observed in 37.5% and 36.8% of mice at 12 weeks of age in PyMT control and PyMT/CCL2 mice, respectively.

### 2.6. The Effect of CCL2 on Early Tumourigenesis in 9 Week Old PyMT Mice

**Figure 5:**
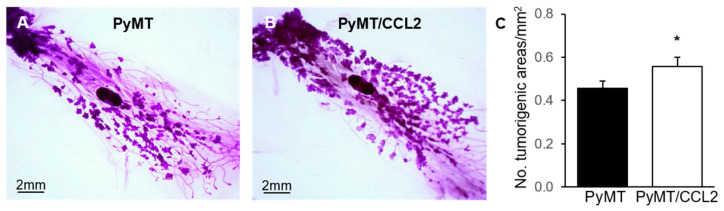
The effect of CCL2 overexpression on early mammary gland tumorigenesis in 9-week-old PyMT transgenic mice. Representative images of carmine alum stained whole mounted 4th pair mammary glands of 9-week-old (**A**) PyMT and (**B**) PyMT/CCL2 mice. (**C**) Tumorigenic areas were measured and analysed as number of tumorigenic areas per mm2 of gland. Data presented as mean + SEM (*n* = 9 per group). Measured data were analysed using a simple linear regression, and number of tumours was analysed using a negative binomial regression with Wald Chi-square post hoc comparison. Statistical significance is indicated by * when *p* < 0.05.

Mice that carry the Mmtv-Pymt transgene spontaneously develop mammary gland tumours from 6 weeks of age. Prior to the emergence of palpable tumours, there are early tumorigenic events such as hyperplasia in this mouse model [41]. We sought to investigate the impact of CCL2 overexpression on very early tumorigenesis. The fourth pair mammary glands, where there were no palpable tumours, were collected from 9-week-old mice and carmine alum stained for whole mount analysis of early tumorigenesis. All dissected mammary glands from both PyMT/CCL2 and PyMT controls exhibited extensive areas of early tumorigenesis at 9 weeks of age (Figure 5A,B). When the number of individual tumorigenic areas per mouse was manually counted, there was a significant increase in the number of tumorigenic areas per mm^2^ of mammary gland for PyMT/CCL2 compared to PyMT controls (0.557 ± 0.04 and 0.455 ± 0.03 areas per mm^2^, respectively (*p* = 0.047) (Figure 5C).

### 2.7. The Effect of CCL2 on Macrophage Infiltration of Mammary Tumours

**Figure 6:**
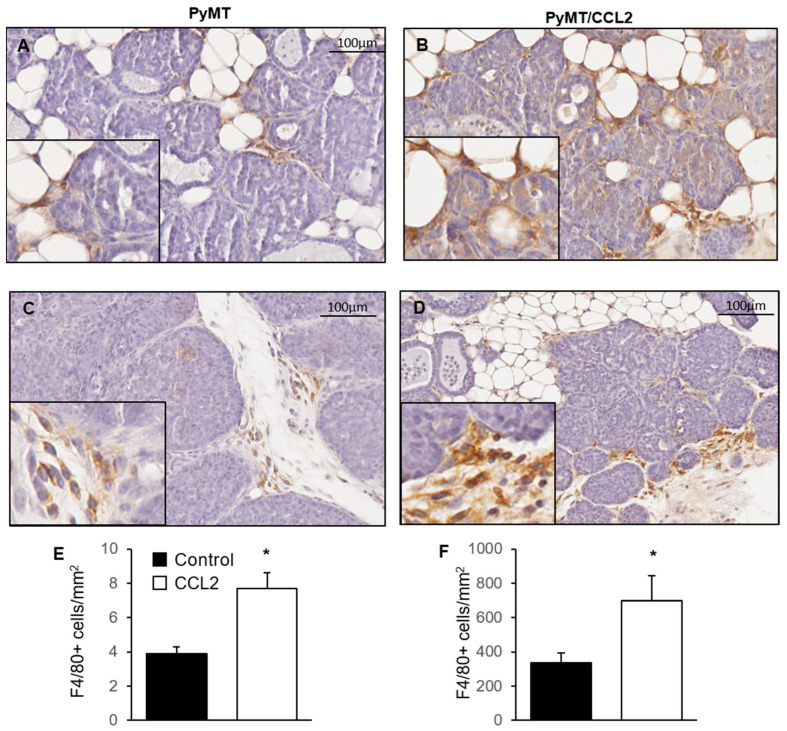
The effect of CCL2 overexpression on macrophage abundance in mammary tumours in 9-week-old PyMT transgenic mice. Representative images of F4/80 stained mammary glands of 9-week-old (**A**,**C**) PyMT and (**B**,**D**) PyMT/CCL2 mice in areas of (**A**,**B**) early tumorigenesis and (**C**,**D**) the primary tumour. Images were taken at 20× magnification with 40× inserts. F4/80+ cells were manually counted in 3 areas of (**E**) early tumorigenesis or (**F**) primary tumour (*n* = 10 per group). Arrows indicate F4/80+ stained cells. Data presented as mean F4/80+ cells per mm + SEM with statistical analysis performed using a simple linear regression with Wald Chi square post hoc comparison. Statistical significance indicated by * when *p* < 0.05.

To investigate the effect of CCL2 on macrophage infiltration during tumorigenesis in the Mmtv-PyMT mouse model, fourth pair mammary glands were dissected at 9 weeks’ age from PyMT and PyMT/CCL2 and formalin fixed paraffin embedded tissue sections were stained with pan macrophage marker F4/80. Three areas of non-palpable early tumorigenesis were randomly identified, with the assessor blinded to mouse genotype, in each mammary gland and the number of F4/80+ cells were manually counted (Figure 6A–D). In PyMT mice, the average number of F4/80 stained macrophages per mm^2^ was 3.87 ± 0.413, and in PyMT/CCL2 mice there was an average of 7.67 ± 0.961 macrophages per mm^2^. There were significantly more macrophages infiltrating early tumorigenic tissue in PyMT/CCL2 mice compared to PyMT controls at 9 weeks of age (*p* < 0.001) (Figure 6E,F). Palpable primary tumours collected from PyMT control mice at 9 weeks of age had an average of 497.54 ± 85.02 macrophages per mm^2^, and PyMT/CCL2 mice had an average of 683.00 ± 97.81 macrophages per mm^2^ (Figure 6B,D,F).

### 2.8. The Effect of CCL2 on Global Gene Expression in the Mammary Gland

**Figure 7:**
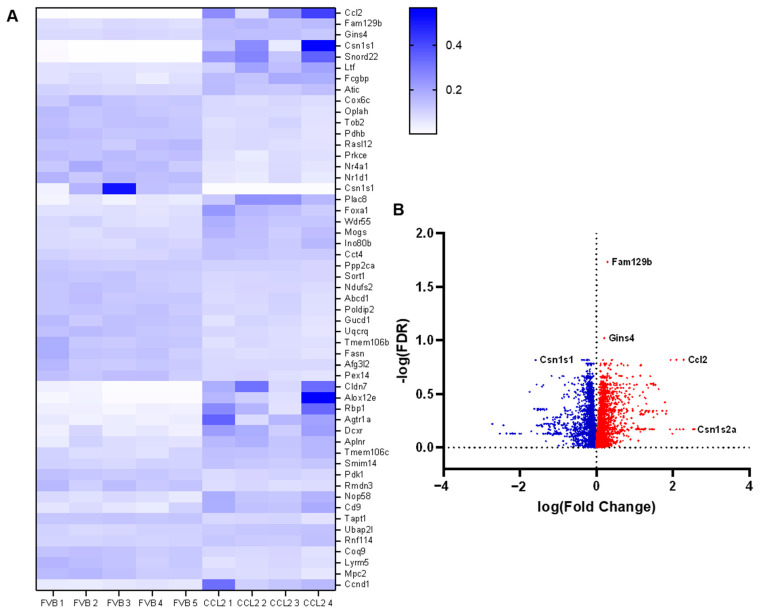
The effect of CCL2 overexpression on macrophage abundance in mammary tumours in 9-week-old PyMT transgenic mice. RNAseq analysis was performed on mammary glands from 12-week-old Mmtv-Ccl2 mice. (**A**) Heat-map depicting relative gene expression of top 50 significant genes in Mmtv-Ccl2 mice versus FVB wild-type controls. (**B**) Volcano plot shows differentially expressed genes according to log(fold change).

To investigate the effect of CCL2 on mammary gland global gene expression, mammary glands from Mmtv-Ccl2 and FVB control mice were collected at 12 weeks of age and analysed for global gene expression by RNAseq. These were healthy normal mammary glands, not tumorigenic, as these mice did not carry the PyMT oncogene. The results of the RNAseq analysis showed 6149 genes that were differentially expressed in mammary glands of Mmtv-Ccl2 mice. The differentially expressed genes are represented by heat-map (top 50 genes) and volcano plot (Figure 7A,B). The most differentially expressed gene between Mmtv-Ccl2 and wild-type mice is CCL2, in agreement with the genotype of the transgenic model.

Of the 6149 genes identified using RNAseq, 2783 genes (45%) were upregulated in mice overexpressing CCL2 in their mammary glands. Functional enrichment analysis of those genes using DAVID revealed cellular pathways significantly upregulated in Mmtv-Ccl2 mouse mammary glands, these pathways are summarised in Table 1. A number of pathways involved in cancer and cell survival were upregulated in Mmtv-Ccl2 mice compared to wildtype controls including proteoglycans in cancer (*p* = 1.39 × 10^−8^), cell cycle (*p* = 7.36 × 10^−5^), pathways in cancer (*p* = 4.4 × 10^−4^), Ras signalling (*p* = 0.0162) and PI3K-Akt signalling (*p* = 4.23 × 10^−4^). In addition, a number of pathways involved in DNA damage and repair were upregulated such as DNA replication (*p* = 5.57 × 10^−5^), mRNA surveillance (*p* = 0.0012), nucleotide excision repair (*p* = 0.0033), mismatch repair (*p* = 0.0102), p53 signalling (*p* = 0.0155) and transcriptional misregulation in cancer (*p* = 0.0425). Other upregulated pathways were involved in the extracellular matrix (*p* = 0.0077) and TGFB signalling (*p* = 0.0389). Endocrine pathways upregulated in Mmtv-Ccl2 mice include prolactin signalling (*p* = 0.0345) and oestrogen signalling pathways. Angiogenic genes involved in VEGF signalling (*p* = 0.051) and leukocyte migration (*p* = 5.35 × 10^−4^) were also upregulated (Table 1).

Of the 6149 genes identified using RNAseq, 3366 genes (55%) were downregulated in mice overexpressing CCL2 in their mammary glands. Functional enrichment analysis using DAVID of those genes revealed cellular pathways that were significantly downregulated in Mmtv-Ccl2 mouse mammary glands, and these pathways are summarised in Table 2. In Mmtv-Ccl2 mouse mammary glands, 386 genes involved in metabolic pathways were downregulated (*p* = 5.83 × 10^−42^). Specific metabolic pathways downregulated in these mice include those involved in glucose metabolism such as citric acid cycle (*p* = 4.54 × 10^−17^), insulin signalling pathway (*p* = 6.58 × 10^−10^), AMPK signalling (*p* = 1.38 × 10^−9^) and glycolysis and gluconeogenesis (*p* = 1.21 × 10^−7^). Fatty acid signalling pathways downregulated included fatty acid metabolism (*p* = 1.26 × 10^−14^), PPAR signalling (*p* = 1.63 × 10^−11^), fatty acid degradation (*p* = 6.55 × 10^−10^), fatty acid elongation (*p* = 2.05 × 10^−6^), fatty acid biosynthesis (*p* = 1.25 × 10^−4^), adipokine signalling (*p* = 1.13 × 10^−6^) and regulation of lipolysis (*p* = 4.84 × 10^−5^). Immune signalling pathways involved in T cell and B cell receptor signalling were also suppressed (*p* = 0.004 and 0.016, respectively).

## 3. Discussion

While the role of macrophages in the mammary gland has been extensively investigated, the interplay between fibroblasts and macrophages and the role of CCL2 in the mammary gland are not well-understood. In this study, we have demonstrated that THP-1-derived macrophages have significant roles in modulating fibroblast gene expression and collagen production, and the crosstalk between mammary fibroblasts and macrophages can influence the macrophage phenotype. Further, this study supports CCL2 as a mediator of these interactions that contributes to mammographic density and early breast cancer tumorigenesis through regulation of ECM, cancer gene pathways, and metabolic signalling.

### 3.1. The Effect of CCL2 on ECM Regulation and Mammographic Density

Our studies report that CCL2 reduces the abundance of soluble collagen 1 in the culture supernatant and increases deposition of insoluble collagen fibres in mammary fibroblasts. CCL2 has been investigated for its involvement in fibrotic diseases, which are characterised by excessive deposition of collagen and other ECM components. CCL2 is upregulated by hepatic monocytes during liver fibrosis, and production of CCL2 is elevated in both the serum and lungs of patients with pulmonary fibrosis [42,43,44]. Mice overexpressing CCL2 in the mammary glands exhibited a greater thickness of stromal collagen in the mammary glands compared to wildtype controls as well as an increase in mRNA expression of ECM genes *Lox* and *Timp3* [11]. Our studies investigated how constitutive expression of CCL2 in the mammary glands of non-tumorigenic mice affected overall gene expression using RNAseq. Pathway analysis of differentially expressed genes revealed that Mmtv-Ccl2 mice have upregulated expression of genes involved in extracellular matrix and collagen deposition at 12 weeks of age. These characteristics are reminiscent of the increased proportion of stroma and collagen deposition in human breast tissue with high mammographic density, where CCL2 expression is observed to be increased in the epithelium of high density breast tissue [11,12]. These results support the notion that CCL2-mediated inflammation may be a driver of high mammographic density.

On the other hand, while many studies have linked CCL2 to fibrosis and excess collagen deposition, some studies have reported that CCL2 can have anti-fibrotic functions. It has also been observed that CCL2 downregulates the expression of collagen 1 in pulmonary fibroblasts [45]. Another study reported impaired resolution of liver fibrosis in CCR2 knockout mice [46]. Other studies have reported no effect of CCL2 on collagen production in fibroblasts [47]. This suggests that the role of CCL2 in collagen production is complex and requires further studies. Overall, these results suggest that CCL2 increases stromal collagen deposition; however, this may be regulated by the surrounding microenvironment. Future studies should further investigate the interaction of CCL2 driven macrophages in modulating the ECM at a protein level through the measurement of hydroxyproline and collagen regulation by LOX.

### 3.2. Mammary Fibroblasts Drive Differentiation of Macrophages

The expression of specific cell surface markers on macrophages is used to describe their polarization to either M1 or M2 phenotype. M1 macrophages express cell surface markers such as CD80, CD11c and are considered to exert largely anti-tumour effects [38,48]. On the other hand, M2 macrophages express markers such as CD163 and CD206 and can exert tumour promoting functions [39,49,50]. Our study of crosstalk between mammary fibroblasts and THP-1-derived macrophages demonstrated that there is an increase in alternatively activated CD163+ M2 macrophages when co-cultured with primary mammary fibroblasts. Further, the co-cultured macrophages exhibited greater expression of inflammatory cytokines *IFNG*, *CCL2* and the CCL2 receptor *CCR2*, as well as angiogenesis growth factor *VEGF*.

Our findings of increased percentage of pro-tumorigenic M2 macrophages with increased expression of inflammatory and angiogenic growth factors when co-cultured with mammary fibroblasts is supported by others. Ferrer et al. co-cultured human dermal fibroblasts with M1 macrophages [51], which promoted the expression of the CD163 surface marker and skewed macrophages to an M2 phenotype. Another study showed that colorectal cancer cells promote the polarization of macrophages to the M2 phenotype characterised by surface marker CD163, mediated by CCL2 and ICAM1 [27]. Previous studies have demonstrated that alternatively activated M2 macrophages have tumour promoting functions, and tumour-associated macrophages (TAMs) have a similar phenotype to M2 macrophages [38,49,52]. One study observed that CD14+ human peripheral blood mononuclear cells (PBMCs), when co-cultured with fibroblasts from pancreatic cancer tissue, exhibited an increased expression of M2 macrophage phenotype markers CD163 and CD206 [35]. Both M2 and TAM subtypes produce growth factors and angiogenesis promoting factors to drive tumour progression such as VEGF and PDGF [53,54,55,56]. The capacity of mammary fibroblasts in polarizing macrophages could be further assessed through additional stimulation of co-cultures with M1 driving LPS and IL4 proteins. The inhibition of M1 polarization would provide additional evidence for this role of mammary fibroblasts.

Alternatively activated macrophages have been implicated in breast cancer. Previous studies have demonstrated that CD163+ macrophages in the tumour stroma of breast cancer tissues correlated to high tumour grade, larger tumour size, triple negative cancers, and suggest that CD68+ macrophages in the tumour stroma is an independent prognostic marker for cancer free survival [56,57]. Our results show that mammary fibroblasts stimulate the polarization of macrophages to an alternatively activated M2 phenotype, expressing a high abundance of inflammatory cytokines and pro-angiogenic factors that could favour tumour development and progression in breast cancer. However, the role of fibroblast-macrophage interactions in mammographic density is still unclear. Previous studies of have reported there to be less CD206+ alternatively activated macrophages in the stroma of high density breast tissue areas compared to low density breast tissue areas [12]. Therefore, further studies are required to elucidate the role of macrophages, and their interactions with mammary fibroblasts in mammographic density and the associated breast cancer risk.

### 3.3. Crosstalk between M2 Polarized Macrophages and Mammary Fibroblasts

Our co-culture studies suggest that a complex interaction occurs between macrophages and fibroblasts, resulting in a pro-inflammatory microenvironment with increased extracellular matrix breakdown and deposition. These interactions between cell types are likely part of the crosstalk necessary for breast tissue function; however, they can also contribute to a microenvironment that supports tumorigenesis. Inflammatory COX2 expression in fibroblasts is known to shape the immune microenvironment in the lung and provide a niche for metastatic breast cancer cells [58]. M2 macrophages have roles in tissue remodelling and are found in fibrotic conditions where there is an abundance of collagen and the extracellular matrix. Our study found that macrophages have higher expressions of *MMP2* and *TIMP1,* and a reduced expression of *MMP9* when co-cultured with mammary fibroblasts. A balance of the expression of MMPs and their inhibitors, TIMPs, are important in the production and degradation of collagen and the extracellular matrix. Previous studies suggest that TIMP1 is a critical mediator of inflammation and M2 macrophage polarization in colorectal cancer [27]. Alternatively activated M2 macrophages can also influence tissue remodelling and collagen production through their effects on fibroblasts, and can promote fibrotic activity of fibroblasts by increasing collagen production and expression of MMPs that remodel the extracellular matrix [32].

Our studies demonstrated that when in co-culture with THP-1-derived macrophages, mammary fibroblasts upregulated matrix remodelling genes *MMP1*, *MMP3*, and *MMP9*. Other studies have reported that stimulation of human dermal fibroblasts with M2 macrophage conditioned media resulted in upregulation of collagen production; however, contradictory to our studies, it was also reported that MMP gene expression was downregulated [30]. Interestingly, expression of MMPs by mammary fibroblasts was upregulated when co-cultured with macrophages, despite the reduction in TGFB observed in these cells. TGFB is an anti-inflammatory cytokine and it would be unusual for it to be upregulated concurrently with increases in inflammatory CCL2, COX2 and IL-6 and -8. Nonetheless, TGFB is often associated with increased extracellular turnover and is known to promote macrophage-mediated mammary tumorigenesis [23]. Our study investigated one TGFB isoform (i.e., *TGFB1*) at the mRNA level, and further studies of other isoforms and secretion of TGFB protein could provide more information of the relationships between TGFB, inflammation and tissue remodelling in this co-culture system. The crosstalk between macrophages and mammary fibroblasts is complex; further studies that delineate macrophage phenotypes, and examine their effects on inflammation tissue remodelling and collagen production would help to gain an understanding how these interactions may contribute to the collagen rich environment in high-density breast tissue.

### 3.4. The Role of CCL2 in Tumour Development and Progression

Our results suggest that CCL2 overexpression in the mammary gland increases the expression of genes involved in cancer. Furthermore, PyMT/CCL2 mice had a greater number of tumorigenic areas per mm^2^ in the mammary gland at 9 weeks, suggesting that CCL2 may promote early mammary tumorigenesis. However, further impacts of CCL2 on tumour development in the Mmtv-PyMT mouse model were not observed. In many previous studies, CCL2 has been implicated in promoting tumour progression and metastasis. Previous studies in our lab have demonstrated that CCL2 overexpression reduces tumour latency and tumour-free survival in a carcinogen-induced mammary tumour model [11]. However, it appears that the role of CCL2 in tumour development and progression is multifaceted. Another study by Li et al. reported that tumour growth was reduced in CCL2 null mutant mice, and anti-CCL2 antibody treatment reduced pulmonary metastasis in mice bearing mammary tumours, supporting the role of CCL2 in tumour promotion [59]. However, this study also reported that CCL2 null mutant mice exhibited an increased metastatic spread, and that treatment with anti-CCL2 antibody during early tumorigenesis resulted in a spike in tumour growth [59]. This suggests that CCL2 may play multiple opposing roles during different stages of tumour development and progression. The role of CCL2 may be dependent on the level of CCL2 production by tumours on surrounding tissues. Previous studies in melanoma have reported that a low concentration of CCL2 promoted the survival of tumour cells, while a high concentration of CCL2 resulted in the destruction of tumour mass and high infiltration of immune cells [60,61].

Although inflammation is one of the hallmarks of cancer, the role of CCL2-mediated inflammation in tumour development and progression is more complex. It is suggested that the pro-tumorigenic effects of CCL2 is a result of the chemoattractant properties of CCL2 in the recruitment of cells that suppress immune surveillance or recruit tumour-associated macrophages (TAMs) to tumour sites [5,55,62]. TAMs then produce several factors to support tumour growth as well as tissue remodelling to promote angiogenesis and metastasis [6,8]. TAMs recruited by CCL2 also play roles in immune surveillance and can inhibit tumour development and progression [63]. Our results showed an increase in the infiltration of macrophages to tumour sites in CCL2 overexpressing mice; however, our studies did not delineate the phenotype or functions of these macrophages. Further, the chemoattractant properties of CCL2 are not exclusive to tumour promoting macrophages. CCL2 also recruits a number of other immune cells such as natural killer cells, immature B cells and CD4+ T cells, through the CCL2 receptor CCR2 and binding of CCL2 to the CCR4 receptor [1,64,65]. The recruitment of these cells to the tumour microenvironment may influence immune surveillance and tumour progression. Studies have reported that CCL2 recruits cytotoxic γδ T lymphocytes in vitro, and to tumour sites in vivo [66,67]. These cells have been reported to have cytotoxic effects against breast cancer cell lines both in vitro and in mouse models [68]. The balance of different cell types recruited to the tumour site by CCL2 may determine the rate of tumour growth and progression.

### 3.5. Constitutive CCL2 Expression Downregulates Fatty Acid Synthesis and Metabolism

Interestingly, our studies observed an overall downregulation of genes involved in fatty acid synthesis and metabolism, particularly fatty acid synthase (*FASN*) in mammary glands with constitutive CCL2 expression. This result was unexpected as there is extensive literature reporting increased fatty acid metabolism during inflammation, and CCL2 expression is associated with diseases with increased fatty acid synthesis and metabolism such as fatty liver disease [69,70,71]. Further, fatty acid synthesis and lipid metabolism increased in breast cancer [72,73]. Increased fatty acid metabolism can play a supporting role in the proliferation and survival of breast cancer cells [74,75]. In particular, fatty acid synthase is highly expressed in breast cancer, and has been used as a predictor of decreased tumour-free survival and is associated with the risk of recurrence in breast cancer [76,77,78,79]. A number of studies have investigated the potential of inhibitors of fatty acid synthase as a potential therapy in cancer [75]. However, the expression of fatty acid synthase is also necessary for normal mammary gland development [80]. Currently, there is no literature that implicates CCL2 in the downregulation of fatty acid metabolism and it is unknown if the physiological function of mammary adipose tissue is affected by CCL2. Further investigation may shed light on the role of CCL2 in fatty acid and lipid metabolism.

### 3.6. Limitations

THP-1 cells are an immortal monocyte cell line derived from acute monocytic leukaemia, and therefore may not reflect the physiological function and responsiveness of macrophages that reside in the mammary gland stroma. Further, as this cell line was being co-cultured with primary fibroblasts from human breast tissue samples, there is a possibility for HLA mismatching to occur, which may influence gene expression and other behaviours of the THP-1 derived cells. Due to the limited amount of human tissues we can obtain, it is not feasible to use human samples with only specific HLA types. A greater sample size of human mammary fibroblasts would be required to ensure there is no HLA mismatching. Our studies found that PMA-treated THP-1 macrophages were differentiated into M2 macrophages when in the presence of mammary fibroblasts, and our study utilised a basic analysis of M2 macrophage phenotype by identifying CD163^+^, CD80^−^ cells. However, M2 macrophages have multiple subtypes including M2a, M2b, M2c and tumour-associated macrophages (TAMs), which all have different functions, making the investigation of crosstalk between mammary fibroblasts and M2 macrophages more complex. Future studies may identify proportions of each M2 subtype in the population to better understand the functional role of M2 macrophages in modulating fibroblast activity. Additionally, the physiological levels of CCL2 secreted by macrophages are largely dependent on the microenvironment, and CCL2 could therefore have altered effects at different doses.

The rate of tumour development in the Mmtv-PyMT tumour model is greatly affected by the background strain of the mice. Previous studies have reported that Mmtv-PyMT mice on an FVB background have a reduced tumour latency and greater incidence of pulmonary metastases compared to mice on a C57B6 background [81,82]. In the current study using mice on the FVB background, primary tumours were first palpable around 7–8 weeks of age, and the tumours dissected when the mice were 9 weeks of age were already at late stage carcinoma. The aggressiveness of this tumour development limited our analysis of the effect of CCL2 on early tumorigenesis and hyperplasia. Backcrossing PyMT/CCL2 mice onto a C57B6 background could reduce the rate of tumour development in the model and enable further examination of the tumorigenesis pathways affected by CCL2 overexpression. Future studies using pharmacological inhibition of CCL2 in both our co-culture and in vivo models could provide insights into the interplay of CCL2, macrophages and fibroblasts in breast tumorigenesis.

## 4. Materials and Methods

### 4.1. Isolation and Culture of Human Primary Mammary Fibroblasts

This study was approved by the Human Ethics Committee at the University of Adelaide and The Queen Elizabeth Hospital (TQEH Ethics Approval #2011120). Between 2011 and 2017, participants undergoing reduction mammoplasty, mastectomy for breast cancer removal, or prophylactic mastectomy donated healthy breast tissue as confirmed by the TQEH pathology department. All participants were between the age of 18 and 70, and were capable of giving informed consent. In the event that a participant was pregnant or currently undergoing chemotherapy, the participant was excluded from the study.

Human breast tissue was manually digested using a tissue knife then enzymatically digested using 100 U/mL hyaluronidase (Sigma-Aldrich, St. Louis, MO, USA; Cat#H3506) and 480 U/mL collagenase (Sigma-Aldrich; Cat#C0130) in Advanced DMEM/F12 medium (Thermo Fisher Scientfic, Waltham, MA, USA; Cat#12634010) overnight for 16–18 h at 37 °C with agitation. The digest was then centrifuged at 80× *g* for 1 min and the top liquefied fat layer carefully removed and discarded. The supernatant containing stromal cells was carefully harvested to not disturb the pellet of undigested tissue, then transferred to a clean Falcon tube and pellet re-suspended and brought to 50 mL with supplemented Advanced DMEM/F12. The product was then centrifuged at 400× *g* for 5 min, supernatant was discarded and the pellet re-suspended in 40 mL of supplemented Advanced DMEM/F12 medium. This was repeated until the supernatant became clear. The stromal cell pellet was treated with red blood cell lysis buffer (BD Bioscience, San Jose, CA, USA; Cat#555899) for 15 min, equilibrated with PBS, and filtered through 40 µm cell strainers (Sigma Aldrich; Cat#CLS431750) to remove debris. The cell suspension was washed 3 times in PBS by centrifugation at 400× *g* for 5 min and the supernatant discarded. The pellet was re-suspended in 8 mL of supplement Advanced DMEM/F12 supplemented with 10% foetal calf serum (FCS) (Thermo Fischer Scientific; Cat#10099141), 10 mM HEPES (Thermo Fisher Scientific; Cat#15630080), 2.5 mg/mL Fungizone (Thermo Fisher Scientific; Cat#15290018), 1× L-Glutamine (Thermo Fisher Scientific; Cat#25030081) and 1× Penicillin/Streptomycin (Thermo Fisher Scientific; Cat#15240062) and cultured at 37 °C in 5% CO_2_. Mammary fibroblasts were confirmed by morphological appearance in culture and production of collagen 1.

### 4.2. Culture of THP-1 Monocytes

The human monocyte cell line THP-1 cells were cultured in RPMI-1640 (Life Technologies; Cat#32404014) supplemented with 10% FCS, 1× penicillin/streptomycin, 1× L-glutamine and 10 mM HEPES at 37 °C in 5% CO_2_. Suspended cells were split by dividing suspension fluid into multiple flasks and fresh media was added every 3 days to maintain a cell density of 0.5–1.0 × 10^6^ cells/mL in T75 flasks.

### 4.3. Indirect Co-Culture of Human Mammary Fibroblasts and Differentiated THP-1 Cells

Mammary fibroblasts were seeded into the base of a 6-well plate at 5 × 10^4^ cells/mL in supplemented DMEM media, and grown to 90% confluence before serum starvation overnight. Separately, 2 mL of THP-1 monocyte cell suspension was added to the internal compartment of 24 mm trans-well with 0.4µm pore polycarbonate membrane (Corning, Corning, NY, USA; Cat#3412) at 0.5 × 10^6^ cells/mL and were activated by 5 ng/mL of phorbol-12-myristate-13-acetate (Abcam, Cambridge, UK; Cat#ab120297) in supplemented RPMI media for 72 h to differentiate THP-1 cells to adherent M0 macrophages. Following removal of PMA, trans-well inserts were then placed into the wells containing mammary fibroblasts (0.5 × 10^6^ cells/mL) and were incubated in supplemented, serum free DMEM. Co-culture studies were compared to macrophage-only or fibroblast-only controls, where the cell type was cultured with base medium only.

### 4.4. Enzyme-Linked Immunosorbent Assay

To assess the impact of CCL2 on fibroblast collagen production, fibroblasts were cultured with or without CCL2 (500 µg/mL) for 72 h. Soluble collagen type I in cell conditioned media was measured using a direct coat enzyme-linked immunosorbent assay (ELISA). Standard curves were developed using purified collagen I derived from human placental collagen (BD Biosciences; Cat#354265). Cell supernatants and standardised samples were added to a 96-well Maxisorp plate (Nunc, Roskilde, Denmark; Cat#423501) at a volume of 100 μL per well and stored at 4 °C overnight. The plate was washed with PBS-Tween 0.05% and blocked using a solution of 2.5% bovine serum albumin (BSA) in PBS for 1 h at room temperature. The primary antibody used was rabbit anti-human collagen I polyclonal antibody (Rockland Immunochemicals, Pottstown, PA, USA; Cat#600-401-103-0.5) and was added to the plate at 0.25 μg/mL in a 5% skim dairy milk solution for 3 h at room temperature with agitation. After washing, the secondary antibody europium-tagged anti-rabbit antibody (Perkin Elmer Life Sciences, Waltham, MA, USA; Cat#AD105) was added at 0.5 μg/mL in 1% BSA/PBS for 1 h at room temperature. After the plate was washed, an enhancement solution (Perkin Elmer Life Sciences, Cat#1244-105) was added for 15 min before measuring fluorescence using the FLUOstar Optima plate reader (BMG Labtech Australia, Mornington, VIC, Australia) at excitation 355 nm and emission 620 nm. The collagen concentration for each sample was determined using the standard curve and expressed in μg/mL.

### 4.5. Sirius Red Analysis of Insoluble Collagen

Sirius red dye was used to determine changes in insoluble collagen deposition. Media were aspirated from fibroblast culture plates and washed in PBS. Cells were fixed for 10 min using 100% ethanol. The plate was washed under running tap water and picric acid-sirius red solution (Sigma Aldrich; Cat#365548, Cat#P6744) was added for 1 h. After the stain was removed, the plate was washed with 0.01 M hydrochloric acid and allowed to air dry. To elute the dye, 0.1 M sodium hydroxide was added to the plate. Absorbance was then measured on the FLUOstar Optima plate reader at 550 nm.

### 4.6. Flow Cytometry of THP-1 Macrophages

Differentiated THP-1 cells were detached from the internal channel of trans-wells with trypsin for 2 min at 37 °C. Cells were harvested and placed in 5 mL FACS polypropylene tubes, and washed twice in FACS wash buffer (PBS, 1% FCS, 20 mM sodium azide), 500× *g* for 5 min. Cells were re-suspended in FACS wash to 1 × 10^6^/mL and blocked in a 1% human serum for 20 min at RT. Cells (1 × 10^5^) were aliquoted into FACS tubes containing antibodies: PE/Cy5 conjugated anti-CD80 (clone 2D10) (Biolegend, San Diego, CA, USA; Cat#305210), FITC conjugated anti-CD68 (clone YI/82A) (Biolegend; Cat#333806), APC-H7 conjugated anti-HLA-DR (clone G46-6) (BD Bioscience; Cat#561358) and Alexa Fluor^®^ 647 conjugated anti-CD163 (clone GHI/61) (BD Bioscience; Cat#562669). Staining was performed at 4 °C for 10 min followed by two washes in wash buffer. Cells were re-suspended in 100 µL of buffer and analysed by flow cytometry using FACS Canto II (BD Biosciences).

### 4.7. Mice

Experiments involving use of animals were approved by the University of Adelaide Animal Ethics Committee and were conducted in accordance with the Australian Code of Practice for the Care and Use of Animals for Scientific Purposes [83]. All mice were housed in specific pathogen free conditions with 12 h light cycling (12 h light, 12 h dark) and temperature controlled at the Laboratory Animal Services Medical School Facility. All breeding was performed under ethics approval M-2015-088, and tumour studies were performed under ethics number M-2017-043. Three breeding colonies of mice were maintained and used for these experiments: Mmtv-Ccl2 mice, Mmtv-Pymt mice, and FVB mice. FVB mice are the background strain of both the Mmtv-Ccl2 and Mmtv-Pymt colonies and were used to provide female breeders for the Mmtv-Pymt colony and as wildtype controls.

#### 4.7.1. Mmtv-Ccl2 Mice

Mmtv-Ccl2 transgenic mice were originally generated through sub-cloning and pronuclear microinjection techniques [11]. This colony is homozygous for the Mmtv-Ccl2 transgene and the background strain is FVB. The mouse mammary tumour virus (MMTV) promoter drives constitutive expression of chemokine ligand 2 (CCL2).

#### 4.7.2. MMTV-PyMT Mice

Mmtv-PyMT transgenic mice carry the polyomavirus middle T antigen (PyMT) oncogene driven by the mouse mammary tumour virus (MMTV) promoter. Males heterozygous for the transgene are mated with non-transgenic FVB mice to produce female mice heterozygous for the transgene. When the transgene is expressed in female mice it causes mammary gland tumours with 100% penetrance.

### 4.8. Generation of PyMT/CCL2 Cohort

Male mice heterozygous for the Mmtv-PyMT transgene were mated with female mice homozygous for the Mmtv-Ccl2 transgene. This mating combination resulted in PyMT/CCL2 double transgenic mice that express both the PyMT oncogene and mammary gland specific CCL2 overexpression. The female offspring were genotyped after weaning at 21 days to detect the PyMT oncogene; all mice from this mating combination are heterozygous for the Mmtv-Ccl2 transgene.

### 4.9. Tumour Detection by Palpation

To detect tumours, female PyMT and PyMT/CCL2 mice were monitored weekly from 6 weeks of age. Mice were restrained by the scruff of the neck by holding firmly between the thumb and forefinger. While holding the mouse upright, each of the 10 mammary glands were gently palpated for tumours. The first three mammary pairs were palpated against the rib cage using the forefinger, while the fourth and fifth pairs were palpated by pinching between the thumb and forefinger. Upon detection, mammary tumours were measured using callipers to approximate tumour volume. Mice were sacrificed at 9 and 12 weeks or when tumour volume exceeded 2000 mm^3^.

### 4.10. Histology and Immunohistochemistry

#### 4.10.1. Tissue Collection, Embedding and Sectioning of Mouse Tissues

At the time of autopsy, fourth pair mammary glands, all tumours (half of the primary tumour) and one lung lobe was dissected and each tumour was weighed. All tissue was fixed overnight in neutral buffered formalin (Sigma Aldrich). Tissue was washed twice in PBS over the following two days before being transferred into 70% ethanol and stored until tissue processing. Tissue was processed using the Excelsior AS Tissue Processor (Thermo Fischer Scientific) with the following dehydration and embedding protocol; 60 min 70% ethanol, 60 min 85% ethanol, 60 min 90% ethanol, 60 min 96% ethanol, 2 × 60 min 100% ethanol, 2 × 60 min Xylene and held in paraffin wax at 62 °C under vacuum conditions until embedding. Tissue was moulded into paraffin wax blocks and stored at room temperature. Tissue sections were cut at 5µm thickness using the Leica Rotary Microtome (Leica Microsystems, Wetzler, Germany) onto SuperFrost Plus Slides. Tissue sections were then bonded onto slides on a 37 °C heating block for 30 min and stored at room temperature for histology.

#### 4.10.2. Mammary Gland Whole Mount Preparation

Upon dissection, the fourth pair mammary glands were collected from PyMT and PyMT/CCL2 mice at 9 weeks of age and spread on a glass slide. Whole mounted mammary glands were fixed for a minimum of 4 h in Carnoy’s fixative (60% ethanol, 30% chloroform and 10% glacial acetic acid). Once fixed, the slides were washed in 70% ethanol for 15 min then rinsed in MilliQ water for 5 min. The slides were stained with carmine alum (2% carmine (Sigma Aldrich; Cat#C1022), 5% aluminium potassium sulphate (Sigma Aldrich; Cat#237086)) overnight. Stained whole mounted mammary glands were washed in 70% ethanol for 15 min, then twice in 100% ethanol for 15 min each. Slides were then places in xylene to be cleared for 1 week before mounting with a coverslip with Entellan mounting media (Proscitech, Adelaide, SA, Australia; Cat#IM022).

#### 4.10.3. Haematoxylin and Eosin Staining

Paraffin embedded mammary gland tissue sections were dewaxed using Xylene (Merck Millipore, Darmstadt, Germany; Cat#108298) for 3 × 5 min, and rehydrated in gradual dilutions of ethanol for 3 min each (2 × 100%, 1 × 90%, 1 × 70% and 1 × 50%), followed by 2 min in MilliQ water. Tissue sections were stained in haematoxylin (Sigma-Aldrich; Cat#HHS16) for 30 s, then stained in eosin (Sigma-Aldrich; Cat#318906) for 10 s. Sections were then dehydrated through gradual increase of ethanol concentration (2 min 90%, 2 × 1 min 100%), and cleared by 2 × 5 min in Xylene. Slides were then mounted with coverslips using Entellan mounting media.

#### 4.10.4. Immunohistochemistry to Detect F4/80

Mouse tissue sections were dewaxed in xylene for 2 × 5 min and rehydrated by graduated dilutions of ethanol (100% ethanol for 3 × 5 min, 90%, 70% and 50% ethanol for 3 min each) then milliQ water for 1 min. Sections were then incubated in a freshly made quench solution to block endogenous peroxidase activity comprised of 10 mL of hydrogen peroxide, 90 mL of water and 100 mL of methanol. Following 3 × 3 min washes in water, the sections were blocked using 15% normal rabbit serum in PBS for 30 min at 37 °C. The primary antibody (rat anti-mouse F4/80 (Ebioscience, San Diego, CA, USA; 14480182)) was added at a 1 in 50 dilution with 1.5% normal rabbit serum, and incubated overnight at 4 °C in a humidified chamber. Sections were washed twice in PBS for 5 min and then incubated with the secondary antibody (biotinylated rabbit anti-rat IgG antibody (Vector Laboratories, Newark, CA, USA; Cat#BA4000)) at a dilution of 1 in 500 for 40 min at room temperature. Slides were washed for 3 × 5 min in PBS and Incubated with the Vectastain ABC Elite Kit (Vector Laboratories; Cat#PK4000) for 30 min, and positive staining was then detected using 3,3′-diaminobenzidine (DAB) (Dako, Santa Clara, CA, USA; Cat#K3468) according to manufacturer’s instructions for 10 min at room temperature. Tissue sections were then counterstained with haematoxylin for 30 s and rinsed in running tap water. Sections were dehydrated through graduated increases in ethanol concentrations (2 × 2 min in 90% ethanol, 2 × 1 min in 100%) and then cleared for 2 × 5 min in xylene. Slides were mounted using DPX mounting media (Merck). Slides stained with secondary antibody only were included as negative controls.

### 4.11. Image Capture and Quantification

#### 4.11.1. Haematoxylin and Eosin Stained Mouse Tissues

Mouse mammary glands, primary tumours and lungs, were collected from PyMT/FVB and PyMT/CCL2 mice at 9 and 12 weeks of age and tissue sections were stained with haematoxylin and eosin. Sections were analysed by veterinary pathologist (L.W.) blinded to mouse genotype for tumour grade, cytological atypia, tumour necrosis and pulmonary metastasis. Mouse mammary gland sections were also assessed for various tumour grade regions across the whole mammary gland at 9 weeks.

#### 4.11.2. Whole Mounted Mammary Glands

Whole mounted mammary glands were imaged using an Olympus SZ61 stereo microscope and an SC50 camera (Olympus, Shinjuku, Tokyo, Japan). Images were de-identified and analysed using Image J Version 1.5.3). The total number of hyperplastic areas were manual counted both distal to the lymph node as well as the entirety of the mammary gland. The total hyperplastic area and percentage hyperplasia were both calculated by measuring the total area of the mammary gland and measuring the areas occupied by hyperplastic tissue.

#### 4.11.3. F4/80 Quantification in Mouse Tumours

F4/80 stained primary tumours and fourth pair mammary glands collected from PyMT and PyMT/CCL2 mice at 9 and 12 weeks stained with F4/80 were captured as digital images using a Nanozoomer digital scanner (Hamamatsu photonics, Shizuoka, Japan) with a zoom equivalent to a 40× objective lens. To quantify F4/80 staining in primary tumours, areas containing positively stained macrophages were measured across the entire section and the number of positively stained cells were manually counted. Data were expressed as an average positive cells/mm^2^ for each section. To quantify F4/80 staining in fourth pair mammary glands at 9 weeks, 3 areas of hyperplasia were identified distal to the lymph node. The number of F4/80 positive cells around the border of these areas was manually counted. Data were expressed as the average positively stained cells/mm. All quantifications were carried out blinded to mouse genotype.

#### 4.11.4. Genotyping Mice

Genotypes of mice were determined using polymerase chain reaction (PCR) using DNA extracted from mouse tail tips. DNA from PyMT and PyMT/CCL2 mice were analysed for presence of the Pymt oncogene. Tail tips were digested in 350 μL of digestion buffer containing 17 mM tris, 17 mM EDTA, 170 mM sodium chloride, 0.85% SDS (PH 7.8) with 0.1 mg proteinase K (all from Sigma Aldrich) at 55 °C overnight. 5 μL of the tail digest was diluted in 95 μL of MilliQ water and incubated for 15 min at 95 °C to inactivate the proteinase K. Extracted genomic DNA was stored at 4 °C until PCR analysis was performed. Presence of the Mmtv-PyMT gene was performed using PCR. The primers to detect the PyMT allele are; forward 5′-GAGCGAGGAACTGAGGAGAG-3′ and reverse 3′-CTTAGGCGGCGACTGGTAGC-5′ with product size 195 base pairs (Geneworks, Thebarton, SA, Australia). The PCR reaction mixture (25 μL) contained 2.5 uL of 10× DNA polymerase reaction buffer (Fisher Biotec, Wembley, WA, Australia; Cat#230924), 2.5 μL of 25 mM MgCl_2_ (Fisher Biotec; Cat#210924), 0.5 μL of 10 mM dNTPs (Roche, Munich, Germany; Cat#1277049), and 1.25 μL of each forward and reverse primer, 0.125 μL of Taq polymerase (Fisher Biotec; Cat#2309090), 16 μL of MilliQ water and 2 μL of sample DNA. The PCR conditions were 5 min at 94 °C for 5 min, followed by 35 cycles of 30 s at 94 °C, 30 s at 55 °C, 1 min at 72 °C and one cycle of 7 min at 72 °C.

PCR products of DNA encoding PyMT genotyping were detected using gel electrophoresis. The PCR products were separated on a 2% agarose gel (Sigma-Aldrich; Cat#A9539) by dissolving agarose in tris-acetate-EDTA (TAE) buffer containing 0.01% GelRed^TM^ (Biotium, Fremont, CA, USA; Cat#41002) as intercalating agents to detect DNA. The PCR products were loaded to the gel with 1× loading buffer and run for 40 min at 80 volts. Gel images were captured using Gel Doc^TM^ EZ Imager (BioRad, Hercules, CA, USA) under UV light. 

#### 4.11.5. RNA Extraction from Frozen Tissues

Third pair mammary glands were collected from Mmtv-Ccl2 and FVB mice at 12 weeks age during metestrus and diestrus, and snap frozen in liquid nitrogen. Tissues were then stored at −80 °C under RNAse-free conditions until use for RNA applications. To extract RNA, these tissues were transferred to a new tube containing 0.6 g of 1.4 mm ceramic beads (QIAGEN, Clayton, VIC, Australia; Cat#13113-325) and 1 mL of Trizol reagent was added (ThermoFisher; Cat#15596026). Tissue was homogenised with the Powerlyser 24 Bench Top Bead Based Homogenizer (MoBio, Carlsbad, CA, USA) at 30 Hz for 3 × 2 min, and repeated if the tissue was still not homogenous. Samples were incubated at room temperature for 5 min, then 200 μL of chloroform (Sigma-Aldrich; Cat#288306-1L) was added and the tubes were shaken vigorously, incubated on ice for 15 min, then centrifuged for 15 min at 11,000× *g* at 4 °C. Approximately 500 µL was collected of the aqueous phase containing RNA, and transferred into a new tube. An equal volume of isopropanol (Sigma-Aldrich; Cat#563935) was added to the tubes and samples were incubated at −20 °C overnight. The following day, samples were centrifuged for 30 min at 11,000× *g* at 4 °C. The RNA pellet was then washed twice with ice cold 70% ethanol and centrifuged for 10 min at 11,000× *g* at 4 °C. The pellet was air dried on ice for 30 min and dissolved in 50 µL of RNase free water. RNA samples were then treated with DNase to remover genomic DNA contamination using the TURBO DNase Kit (Invitrogen; Cat#AM1907). According to the manufacturer’s instructions, 5 µL of 10× TURBO DNase Buffer and 1 µL of TURBO DNase was added to each sample, then incubated at 37 °C for 30 min. To deactivate the DNase enzyme, 5 µL of the DNase inactivation reagent was added to each sample and continuously mixed for 5 min at room temperature. Samples were then centrifuged at 10,000× *g* for 1.5 min at 4 °C and transferred the RNA into a fresh tube. DNase treated RNA was then quantified using 2 µL of sample on the spectrophotometer ND 1000 Nanodrop using the program ND-1000 V3.7.1. Reverse transcription was performed on 500 ng^−1^ µg of RNA using the iScript cDNA synthesis kit according to manufacturers instructions (Bio-Rad, South Granville, NSW, Australia; Cat#1708890).

#### 4.11.6. Next Generation Sequencing of Mmtv-Ccl2 Mammary Glands

Total RNA was converted to strand specific Illumina compatible sequencing libraries using a NEXTflex Rapid Directional mRNA-Seq library kit (BIOO Scientific, Austin, TX, USA; Cat#NOVA-5138-07) as per the manufacturer’s instructions (v14.10). Briefly, 400 ng of total RNA was polyA selected and the mRNA fragmented prior to reverse transcription and second strand cDNA synthesis using dUTP. The resultant cDNA is poly adenylated before the ligation of Illumina-compatible barcoded sequencing adapters. The cDNA libraries were treated with UDG to degrade the second strand and PCR amplified for 14 cycles prior to assessment by Bioanalyzer (Agilent, Santa Clara, CA, USA) for quality and Qubit fluorescence assay for amount. Sequencing pools were generated by mixing equimolar amounts of compatible sample libraries based on the Qubit measurements. Sequencing of the library pool was performed with an Illumina Nextseq 500 using single read 75 bp v2 sequencing chemistry. Functional gene enrichment analysis was performed on differentially expressed genes, and altered gene pathways were identified using the Database for Annotation, Visualisation and Integrated Discovery (DAVID, Version 6.8) [84,85].

### 4.12. Statistical Analysis

Statistical analysis was performed using SPSS software, version 20.0 for Windows (SPSS, Chicago, IL, USA) in consultation with a statistician (Ms Suzanne Edwards, Adelaide Health Technology Assessment, University of Adelaide). Data were considered statistically significant when *p* < 0.05. An asterisk (*) identified a result that is statistically significant from the control.

All cell culture data, including RT-PCR and collagen production experiments, are presented as mean ± SEM (standard error of mean). Statistical analysis of RT-PCR data was performed using ΔCT values. These data were analysed using a Linear Mixed-effects Model to account for variables such as patient density and treatment groups. Tukey’s post hoc comparisons were performed to determine statistically significant differences in mRNA expression or collagen production between patients with high or low mammographic density, and then between cells treated with immune regulatory proteins and untreated controls.

Kaplan–Meier survival analysis was performed to determine differences in tumour latency between PyMT and PyMT/CCL2 in studies described in Chapter 5 with LogRank test to compare survival curves of each group. Data on tumorigenic areas at 9 weeks of age, tumour burden, primary tumour weigh and quantification of F4/80 stained mammary glands and tumours at 9 and 12 weeks of age were presented as mean ± SEM. Analyses were performed using Linear Regression Models with Post hoc Wald Chi-square comparisons to determine significance. Data from mice at 9 and 12 week of age were analysed separately. Count data including number of tumorigenic areas at 9 weeks and number of tumours at 12 weeks were analysed using a Negative Binomial Regression Model with Wald Chi-square post hoc comparison. Analysis of primary mammary tumours such as tumour grade, cytological atypia, tumour necrosis and lung metastasis at 12 weeks of age were presented as percentage proportions stratified by mouse groups PyMT and PyMT/CCL2. Comparison of proportions were analysed using cross tabulations with Fischer’s exact test.

## 5. Conclusions

In this study, we report that interactions between macrophages and fibroblasts in the mammary microenvironment drive regulation of the extracellular matrix and macrophage phenotype to support tumorigenesis. Further, these interactions may be mediated by CCL2. CCL2 overexpression in the mammary gland increases expression of genes involved in cancer development and progression, and in collagen synthesis. CCL2 increased the infiltration of macrophages and early tumorigenesis. Our results, combined with the literature, support the notion that CCL2 may be a driver of mammographic density and early tumour development.

## Figures and Tables

**Table 1:** Pathway enrichment analysis of upregulated genes from RNAseq analysis of 12-week-old Mmtv-Ccl2 transgenic mouse mammary glands.

KEGG Pathway	Gene Count	*p* Value
Proteoglycans in Cancer	62	1.39 × 10^−8^
DNA Replication	16	5.57 × 10^−5^
Cell Cycle	36	7.36 × 10^−5^
PI3K-Akt Signalling Pathway	76	4.23 × 10^−4^
Pathways in Cancer	84	4.40 × 10^−4^
Leukocyte Migration	33	5.35 × 10^−4^
mRNA Surveillance	27	0.0012
Nucleotide Excision Repair	15	0.0033
ECM Receptor Interaction	23	0.0077
Mismatch Repair	9	0.0102
Oestrogen Signalling Pathway	24	0.0143
P53 Signalling Pathway	18	0.0155
Ras Signalling Pathway	47	0.0162
cAMP Signalling Pathway	41	0.0201
Prolactin Signalling Pathway	18	0.0345
TGFB Signalling Pathway	20	0.0389
Transcriptional Misregulation in Cancer	34	0.0425
VEGF Signalling Pathway	15	0.051

**Table 2:** Pathway enrichment analysis of downregulated genes from RNAseq analysis of 12-week-old Mmtv-Ccl2 transgenic mouse mammary glands.

KEGG Pathway	Gene Count	*p* Value
Metabolic Pathways	386	5.82 × 10^−42^
Non-Alcoholic Fatty Liver Disease	88	1.71 × 10^−29^
Citric Acid Cycle	28	4.54 × 10^−17^
Fatty Acid Metabolism	34	1.26 × 10^−14^
PPAR Signalling Pathway	40	1.63 × 10^−11^
Fatty Acid Degradation	28	6.55 × 10^−10^
Insulin Signalling Pathway	54	6.58 × 10^−10^
AMPK Signalling Pathway	50	1.38 × 10^−9^
Glycolysis/Gluconeogenesis	30	1.21 × 10^−7^
Adipokine Signalling Pathway	30	1.13 × 10^−6^
Fatty Acid Elongation	16	2.05 × 10^−6^
Regulation of Lipolysis in Adipocytes	23	4.84 × 10^−5^
Biosynthesis of Unsaturated Fatty Acids	14	1.25 × 10^−4^
T Cell Receptor Signalling Pathway	29	0.004
B Cell Receptor Signalling Pathway	20	0.016
Fatty Acid Biosynthesis	7	0.018

## Data Availability

Data are available upon request.
